# Physics-Informed Modeling and Control of Multi-Actuator Soft Catheter Robots

**DOI:** 10.3389/frobt.2021.772628

**Published:** 2022-01-14

**Authors:** Seyede Fatemeh Ghoreishi, Ryan D. Sochol, Dheeraj Gandhi, Axel Krieger, Mark Fuge

**Affiliations:** ^1^ Department of Civil and Environmental Engineering and Khoury College of Computer Sciences, Northeastern University, Boston, MA, United States; ^2^ Department of Mechanical Engineering, University of Maryland, College Park, MD, United States; ^3^ Department of Diagnostic Radiology and Nuclear Medicine, School of Medicine, University of Maryland, Baltimore, MD, United States; ^4^ Department of Mechanical Engineering, Johns Hopkins University, Baltimore, MD, United States

**Keywords:** modeling, control, soft catheters, multi-actuator, cerebral aneurysm

## Abstract

Catheter-based endovascular interventional procedures have become increasingly popular in recent years as they are less invasive and patients spend less time in the hospital with less recovery time and less pain. These advantages have led to a significant growth in the number of procedures that are performed annually. However, it is still challenging to position a catheter in a target vessel branch within the highly complicated and delicate vascular structure. In fact, vessel tortuosity and angulation, which cause difficulties in catheterization and reaching the target site, have been reported as the main causes of failure in endovascular procedures. Maneuverability of a catheter for intravascular navigation is a key to reaching the target area; ability of a catheter to move within the target vessel during trajectory tracking thus affects to a great extent the length and success of the procedure. To address this issue, this paper models soft catheter robots with multiple actuators and provides a time-dependent model for characterizing the dynamics of multi-actuator soft catheter robots. Built on this model, an efficient and scalable optimization-based framework is developed for guiding the catheter to pass through arteries and reach the target where an aneurysm is located. The proposed framework models the deflection of the multi-actuator soft catheter robot and develops a control strategy for movement of catheter along a desired trajectory. This provides a simulation-based framework for selection of catheters prior to endovascular catheterization procedures, assuring that given a fixed design, the catheter is able to reach the target location. The results demonstrate the benefits that can be achieved by design and control of catheters with multiple number of actuators for navigation into small vessels.

## 1 Introduction

**FIGURE 1:**
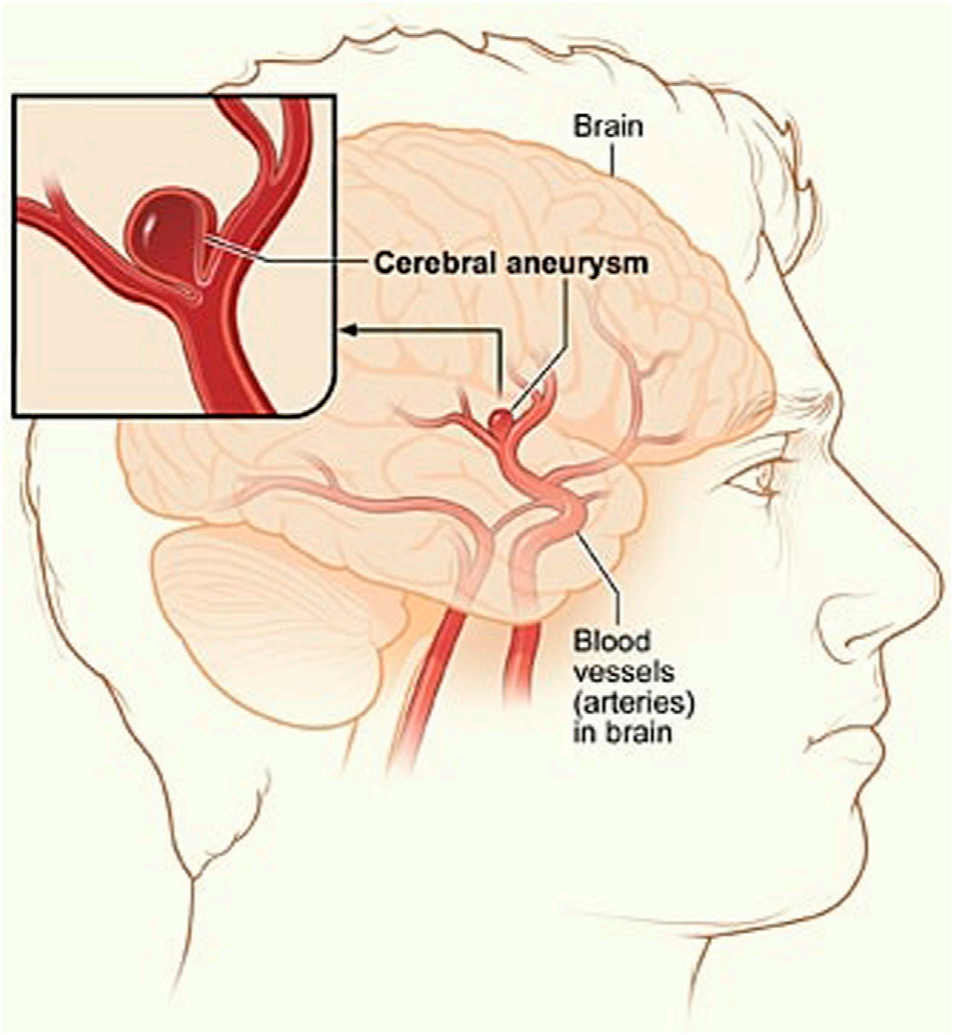
A depiction of cerebral aneurysm adapted from Ref. You et al. (2017).

A cerebral aneurysm is a weak or thin spot on an artery in the brain that bulges outwards like a balloon due to the pressure of the blood being pumped through the artery Brisman et al. (2006). An example of cerebral aneurysm is shown in Figure 1.

The bulging aneurysm can put pressure on the nerves or brain tissue and it has the potential to rupture and cause bleeding within the brain or surrounding area. This leads to an extremely serious condition known as a haemorrhage, which can cause extensive health problems such as stroke, brain damage, coma, and even death. Approximately 12 percent of patients die before receiving medical attention Schievink (1997). Therefore, early detection and treatment of a brain aneurysm before its rupture is critical for saving lives of a large group of patients suffering from this issue Wiebers et al. (2003).

The ultimate goal of aneurysm treatment is to exclude the aneurysmal sac from the intracranial circulation while preserving the parent artery. Treatment of cerebral aneurysms has long been the domain of neurosurgeons performing open brain surgeries, but since 1990, neuroradiologists have been using minimally invasive procedures to treat increasing numbers of patients with cerebral aneurysms Schievink (1997). Open brain surgery or surgical clipping is done by removing part of the skull to reach the aneurysm. The surgeon places a metal clip across the neck of the aneurysm to prevent blood flow into the aneurysm bulge. Once the clipping is done, the skull is closed back together. In minimally invasive procedure or endovascular coiling, also called coil embolization, a catheter is inserted into a blood vessel, typically the femoral artery, and passed through blood vessels into the cerebral circulation and the aneurysm. Once the catheter is in place, tiny platinum coils are put through the catheter into the aneurysm to block it and to reduce the flow of blood into the aneurysm. Open brain surgeries carry some risks such as possible damage to other tissue and blood vessels, the potential for aneurysm recurrence and rebleeding, and a risk of stroke Hunt and Hess (1968). However, in minimally invasive procedures, an incision in the skull is not required to treat the brain aneurysm, which leads to decreased morbidity and shorter recovery time. These factors make the catheter-based diagnosis and therapy in minimally invasive procedures a better option over open brain surgeries.

The major cause of unsuccessful catheterization in minimally invasive procedures is the use of conventional micro-catheters which provide very limited maneuverability Hu et al. (2018). The arteries through which the catheter passes are extremely complex and delicate, and navigating to desired intravascular locations in particular can be frequently challenging due to factors such as vascular tortuosity, distal location, vascular stiffness, and numerous side branches arising from larger vessels Kearney and Shabot (1995). The traditional endovascular catheterization is usually performed manually by an interventionalist by push/pull or twisting of catheters with pre-bent tips designated for different anatomical structures. The procedure of manual insertion of a catheter is very risky and challenging as it relies on the skill and experience of the interventionalist. The repeated insertion of a catheter through several trials by the interventionalist could tear a blood vessel at a junction and cause bleeding. The high flexibility of the catheter makes the catheter guidance through the constrained environment very challenging. This task gets more difficult when manipulating the catheter tip through sharp turns where the catheter needs to enter a branch vessel with an origin different from the parent vessel. The reason for the complexity is that the torque transmission to the distal end is inaccurate and limited by frictional forces acting along the catheter length Jayender et al. (2009). Therefore, the interventionalist cannot precisely apply forces on the catheter tip which can sometimes lead to damage to the blood vessel. In addition, the pre-bent shape of the catheter or guidewire can change during the long and tortuous vascular environments. The limited maneuverability and the difficulties in steering and control of the catheters increase the risk for complications including vascular dissection, perforation, and thrombosis. Therefore, there is a need to develop a strategy that can plan and perform catheterization to minimize misplacement and to reduce both risks and patient suffering.

This paper addresses the limited maneuverability of conventional micro-catheters and the challenges of control and actuation by deriving a dynamic model for soft catheter robots with multiple pneumatic actuators. Soft actuators are made of soft and compliant materials such as polymer/metal composites Shahinpoor and Kim (2001); Moghadam et al. (2014); Amiri Moghadam et al. (2015), elastomers Ilievski et al. (2011); Mosadegh et al. (2014), and hydrogels Ionov (2014); Banerjee and Ren (2017). These soft machines operate based on pneumatic Mosadegh et al. (2014); Yang et al. (2015); Agarwal et al. (2016), electrical Shahinpoor and Kim (2001); Moghadam et al. (2015); Amiri Moghadam et al. (2016), chemical Wehner et al. (2016); Zhao et al. (2014), and optical Wie et al. (2016) actuation mechanisms. Of these, soft pneumatic actuators are particularly interesting due to their low cost, simple operation, and relatively long lifetimes Amiri Moghadam et al. (2018). Soft pneumatic actuators are commonly used in soft robotics Lipson (2014); Wehner et al. (2014). Generally, soft pneumatic actuators comprise a series of interconnected inflatable chambers, which are made from elastomers, fabrics, or a combination of both types of these materials. The geometry and material properties of these chambers dictate the motion of the pneumatic actuators, on actuation. These actuators are actuated by the use of compressed air for exerting moments on the catheter to cause bending of the catheter robot. In particular, the air pressure built within their chambers results in their deflection from a straight to a bent configuration.

We model the catheter using Euler–Bernoulli beam theory Moseley et al. (2016), considering the physics of the system as a continuum mechanism with infinite degrees of freedom. We propose a time-dependent model capable of capturing the position of catheter along the vasculature. Using the proposed dynamical model, this paper models the minimally invasive procedure as guiding the multi-actuated soft catheter along a predefined desired trajectory obtained by incorporating the anatomical information and implementing segmentation of pre-operative images and geometric data often available through CT scanning of the brain prior to the interventional procedures. The proposed framework formulates the navigation of multi-actuator soft catheter robot as a constrained optimization problem. The solution to this optimization problem sets the sequence of moments to be exerted by the actuators as well as the insertion depth needed to move the multi-actuator soft catheter along the desired trajectory in such a way that the stress on the vessel wall is minimized. The high performance of the proposed framework in terms of accuracy and speed is demonstrated through a comprehensive set of numerical experiments.

The proposed framework provides a collaborative robotic catheterization within different anatomical geometries by independent control of insertion and bending moments. It develops a simulation-based strategy for selection of the catheter with proper number of actuators prior to endovascular catheterization procedures. The proposed multi-actuator catheter robots can significantly contribute to the success of complex catheterization procedures, allowing surgeons to access the areas of endovascular system that could not be reached with conventional catheters. In addition, by selecting the number of actuators prior to procedures, the number of insertions and retractions normally used by clinicians to guide the catheter correctly into a desired branch to reach the aneurysm location can be reduced considerably, thereby preventing or reducing the possibility of damage to the arteries during the endovascular catheterization procedures. Moreover, the correct placement and actuation of actuators are not always intuitive or simple. Therefore, this computational framework allows the interventionalist to determine the optimal length of catheter insertion and the necessary bending moments. This greatly improves the interventionalist’s ability to control the amount of moments being exerted by actuators while inserting the catheter to precisely follow a predefined trajectory. These deliver the promise of higher accuracy and shorter duration when compared to current catheter-based therapies that depend on the interventionalist’s intuition in guiding the catheter, combating the complications in catheterization procedures.

The rest of the paper is organized as follows: Section 2 presents background on catheterization for endovascular treatment of cerebral aneurysms. The proposed framework is discussed in Section 3, including the formulation for deflection, dynamic modeling, and trajectory tracking of multi-actuator soft catheter robots. Numerical experiments are presented in section 4. Finally, conclusions are drawn and future work opportunities are described in Section 5.

## 2 Background

Endovascular treatment of cerebral aneurysms is expected to benefit from current research and developments in the field of minimally invasive procedure and therapy. Robot-assisted and computer-assisted catheterization methods are the promising approaches to facilitate this medical operation Rafii-Tari et al. (2014). Minimizing the invasiveness of the catheterization process requires catheters capable of moving within the target vessel during trajectory tracking. Furthermore, endovascular procedure outcomes depend greatly on the correct positioning of the catheter. Trajectory tracking and catheter localization require sufficient information about the system dynamics, e.g., a force-deflection relationship of an ablation catheter, or a current-field map in the case of an electromagnetic passive actuation system. Kinematic models for soft robots have been developed based on finite element Duriez et al. (2006); Lenoir et al. (2006), deformation energy Tunay (2004), beam theory Khoshnam et al. (2012), piecewise constant curvature Webster and Jones (2010), and Cosserat rod theory Kratchman et al. (2016). Finite element and energy based methods can be used to predict the robots deformation with great accuracy. However, their high computational cost can hardly meet the required efficiency in kinematics applications Goury and Duriez (2018). The Cosserat rod model is an accurate model. However, the necessity for solving nonlinear differential equations with initial boundary values numerically with no closed-form solution makes this model less attractive. To improve the computational speed, Ref. Tang et al. (2012) explicitly models flexible tips using a less expensive generalized bending model that is more computational efficient than the elastic Cosserat rod model for the slender body. It also describes the simulation algorithms with the use of a minimum coordinates formulation Bergou et al. (2008) to achieve stable and real-time computation. Constant curvature modeling is an accurate approach assuming that external loads are negligible. Constant curvature modeling has been widely used in soft robotics due to the simplifications it enables in kinematic modeling Webster and Jones (2010). Constant curvature robots can be considered as consisting of a finite number of curvatures described by a finite set of arc parameters, which can be converted to analytical frame transformations Robinson and Davies (1999). Therefore, constant curvature can facilitate additional analysis on topics such as design, real-time control, and other useful computations. In case of a constant moment being applied along a beam, Euler-Bernoulli beam mechanics defines a constant curvature result Beléndez et al. (2002); Camarillo et al. (2008).

The broad range of applications of catheterization within minimally invasive procedures demands additional improvements of surgical instruments. Remote-controlled catheters which use Magnetic Resonance Imaging (MRI) for remote steering and guidance have been a field of intensive research since the 1990s Heunis et al. (2020); Sitti (2009); Hwang et al. (2020); Fang et al. (2021). These catheters are equipped with a set of orthogonal coils and magnetic moments generated by the coils deflect the catheter under Magnetic Resonance (MR) magnetic field using Lorentz force. Ref. Greigarn and Cavusoglu (2014) presents a motion-planning algorithm which calculates a sequence of magnetic moments needed to move the tip of the MRI-actuated catheter along a predefined trajectory on a surface. Ref. Zhou et al. (2021) presents a ferromagnetic soft catheter robot system capable of *in situ* computer-controlled bioprinting in a minimally invasive manner based on magnetic actuation. Ref. Roberts et al. (2002) exploits the high magnetic field environment of a clinical MRI scanner and demonstrates the technical feasibility of developing a catheter whose tip can be remotely oriented within the magnetic field by applying a DC current to a coil wound around the catheter tip to generate a magnetic moment and consequent deflection. However, the clinical applicability of the method has been failed to address several practical problems due to the dependence of catheter tip deflection on the initial position relative to the external magnetic field. Furthermore, the catheter torqueability is reduced because of the large axial coil that is needed to attain acceptable catheter deflections. This results in increased heat generation leading to potentially dangerous temperatures at the catheter tip due to high dc currents that need to be applied to the coil.

Ref. Slade et al. (2017) presents a soft catheter capable of apical extension to travel inside constrained environments with minimal shear force. However, the angle and radii of curvature of the trajectories that the designed catheter can track, are limited to specific ranges. A number of methods have been studied to achieve precise and effective positioning of the catheter tip. In Ref. Li et al. (2016), a method is developed to control the tip position using a vertebra-like outer tube with constraint inner tube, but this method requires too big catheter diameters for some surgical purposes. There are also studies that investigated incorporating shape memory alloys into the catheter to control the tip position Fukuda et al. (1994); Hadi et al. (2016). However, using shape memory alloys can cause heating and restrict the range of catheter tip movement considerably, and requires major considerations for safe heating and efficient cooling Fukuda et al. (1994).

Despite several research and development efforts in catheter-based therapies, limited research has been conducted for pre-operative selection of catheters with desired level of maneuverability. The arteries through which the catheter passes are extremely complex and delicate, making the catheterization process a very challenging task. The repeated insertion of a catheter through several trials could tear a blood vessel at a junction and cause bleeding Abreu et al. (2004). Therefore, there is a demand for development of advanced catheters which allow interventionalists access to areas of vascular systems that cannot be reached with conventional catheters. Ref. Gopesh et al. (2021) overcomes this problem with submillimeter diameter, hydraulically actuated hyperelastic polymer devices at the distal tip of microcatheters to enable active steerability. In our previous work Ghoreishi et al. (2021), we proposed the design of soft catheters with multiple actuators, capable of alignment with desired vessel shapes near the target area, and developed a static analytical model for catheter deformation. In this work, we focus on dynamic modeling and control of multi-actuator soft catheters and given a fixed set of designs (i.e., catheters with different geometric and material properties and different number of actuators, etc.), we want to decide prior to catheter-based surgeries that which of these designs is appropriate (capable of tracking) for a desired trajectory leading to the aneurysm. This can advance the future generation of autonomous or semi-autonomous robotic catheterization systems in terms of time, cost, and accuracy, reducing the cognitive workload of the interventionalist while improving the quality of the catheter insertion.

## 3 Proposed Framework

This section describes our proposed strategy for pre-operative selection of the catheter with proper maneuverability, capable of tracking a desired trajectory to reach the aneurysm’s location in the brain. The desired maneuverability is achieved by considering multiple pneumatic actuators along the circular catheter tube. In the following subsections, the proposed formulations for motion modeling and trajectory tracking of multi-actuator soft catheters are presented.

### 3.1 Deflection Formulation of Multi-Actuator Soft Catheter

**FIGURE 2:**
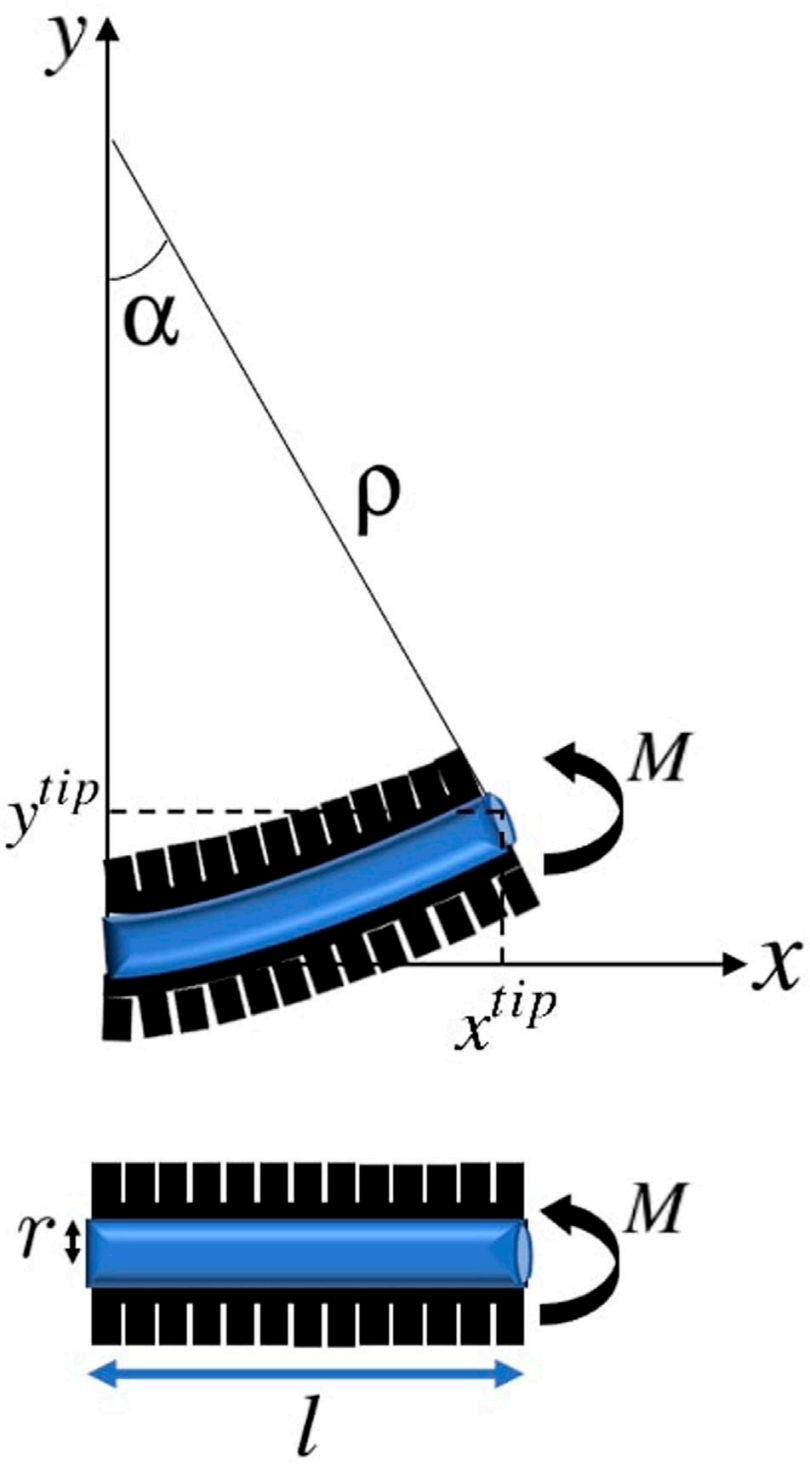
Actuated soft catheter and its deflection under the moment corresponding to the pneumatic actuator, resulting in a circular configuration with the constant radius *ρ* and bending angle *α*.

We consider a soft catheter with a single pneumatic actuator along the catheter’s length *l*, where increasing the internal pressure in the actuator induces a bending moment *M* at the centroid leading to deflection as shown in Figure 2.

As it can be seen, when the actuator is in action, the catheter is in the form of a cantilevered beam. According to the Euler-Bernoulli theorem Moseley et al. (2016), under static conditions, the radius of curvature of the catheter is determined by the bending moment *M* applied by the actuator and the catheter’s Young’s modulus of elasticity *E* and area moment of inertia *I*, as:

$$\rho =\frac{EI}{M}.$$ (1)

Assuming that the catheter bends into a constant curvature shape, the bending angle *α* ^*s*^ corresponding to a point with distance *l* ^*s*^ from the initial point of the catheter is equal to:

$$\alpha^{s}=\frac{l^{s}}{\rho }=\frac{Ml^{s}}{EI}.$$ (2)

Therefore, the horizontal and vertical coordinates of the position of the point with distance *l* ^*s*^ from the initial point of the catheter under moment *M* can be obtained as:

$$\begin{array}{cc} & x\left(M,l^{s}\right)=\rho \;\;\sin\alpha^{s}=\frac{EI}{M}\;\;\sin\left(\frac{Ml^{s}}{EI}\right),\\ & y\left(M,l^{s}\right)=\rho \;\left(1-\cos\alpha^{s}\right)=\frac{EI}{M}\;\left(1-\cos\left(\frac{Ml^{s}}{EI}\right)\right). \end{array}$$ (3)

Clearly, the coordinates of the tip of catheter **p** ^*tip*^ = [*x* ^*tip*^, *y* ^*tip*^] can be achieved by setting *l* ^*s*^ = *l*.

**FIGURE 3:**
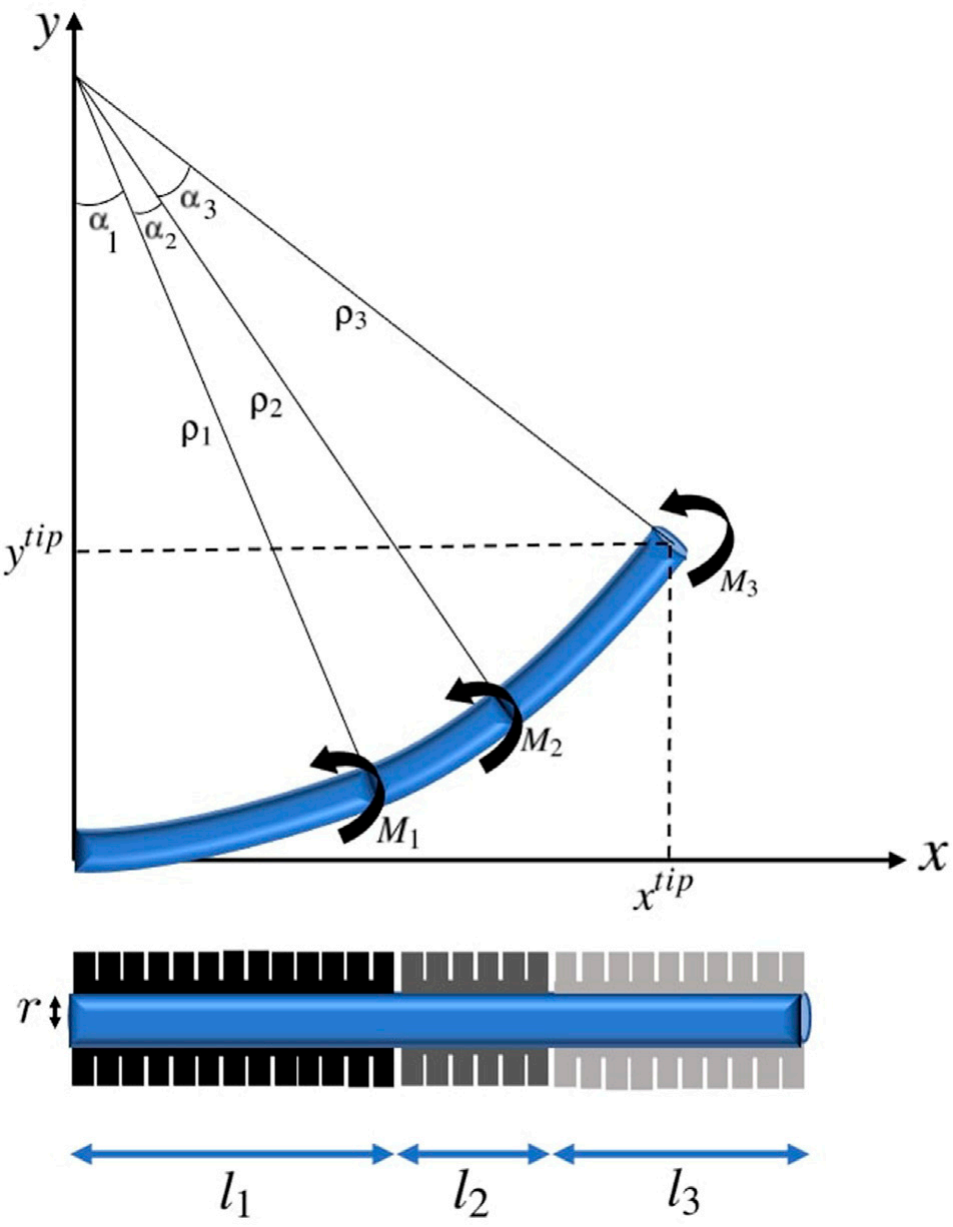
A three-actuator soft catheter and its deflection under the moments corresponding to the pneumatic actuators.

Considering *n* actuators with length *l*_*i*_ along the catheter, the position of any point with distance *l* ^*s*^ from the initial point of the catheter which is with distance $l_{i}^{s}$ from the initial point of the *i*th actuator, i.e. $l^{s}=\sum_{j=1}^{i-1}l_{j}+l_{i}^{s}$ , after some algebraic manipulations, can be obtained as:

$$x\left(M,l^{s}\right)=\overset{i-1}{\underset{j=1}{\sum }}\left[\left(\frac{EI}{M_{j}}-\frac{EI}{M_{j+1}}\right)\;\;\sin\left(\overset{j}{\underset{c=1}{\sum }}\frac{M_{c}\;l_{c}}{EI}\right)\right]+\frac{EI}{M_{i}}\sin\left(\frac{M_{i}\;l_{i}^{s}}{EI}+\overset{i-1}{\underset{j=1}{\sum }}\frac{M_{j}\;l_{j}}{EI}\right),$$ (4)

$$y\left(M,l^{s}\right)=\frac{EI}{M_{1}}+\overset{i-1}{\underset{j=1}{\sum }}\left[\left(\frac{EI}{M_{j+1}}-\frac{EI}{M_{j}}\right)\;\;\cos\left(\overset{j}{\underset{c=1}{\sum }}\frac{M_{c}\;l_{c}}{EI}\right)\right]-\frac{EI}{M_{i}}\cos\left(\frac{M_{i}\;l_{i}^{s}}{EI}+\overset{i-1}{\underset{j=1}{\sum }}\frac{M_{j}\;l_{j}}{EI}\right),$$ (5)

where **M** = [*M*_1_, *…* , *M*_*n*_] denoting the moments applied by *n* actuators. The position of the tip of catheter **p** ^*tip*^ = [*x* ^*tip*^, *y* ^*tip*^] in this *n*-actuator soft catheter can be obtained by setting *l* ^*s*^ = *l* which means $l_{i}^{s}=l_{n}$ . A depiction of a soft catheter with three actuators is presented in Figure 3. Note that the moments exerted by actuators can be in different directions to obtain a S-curve shape.

Hence, the catheter tube is assumed as a circular arc implied by the Bernoulli-Euler beam mechanics and constant independent moments associated with actuators are applied along the catheter. The geometric and material properties along the catheter are assumed to be homogeneous. Specifically *E*, modulus of elasticity, and *I*, moment of inertia of cross section around the natural axis, are fixed along the catheter. Furthermore, the clamping effects are neglected, and the catheter is considered as an elastica, which is a homogeneous unshearable and inextensible slender medium. Therefore the length of the catheter robot does not change as a result of the applied loads. Here, static motion modeling of multi-actuator soft catheter is considered, where only the moments exerted by each of the actuators control the positioning of the catheter. In the next subsection, the dynamic modeling of multi-actuator soft catheter by considering the catheter movement along a desired trajectory is discussed.

### 3.2 Dynamic Modeling and Formulation of Multi-Actuator Soft Catheter

The problem that we aim to address in this work is computation of the sequence of actions that guide the catheter to the target location in a given trajectory. This trajectory is often generated according to the available preoperative geometry of the vessel, which is obtained through preoperative CT/MR scans Zhou et al. (2016). Vascular centerlines are typically used as a reference trajectory, with researchers studying skeletonization of pre-operative images for extracting blood vessel shapes and centerlines Cheng et al. (2012); Li et al. (2021).

**FIGURE 4:**
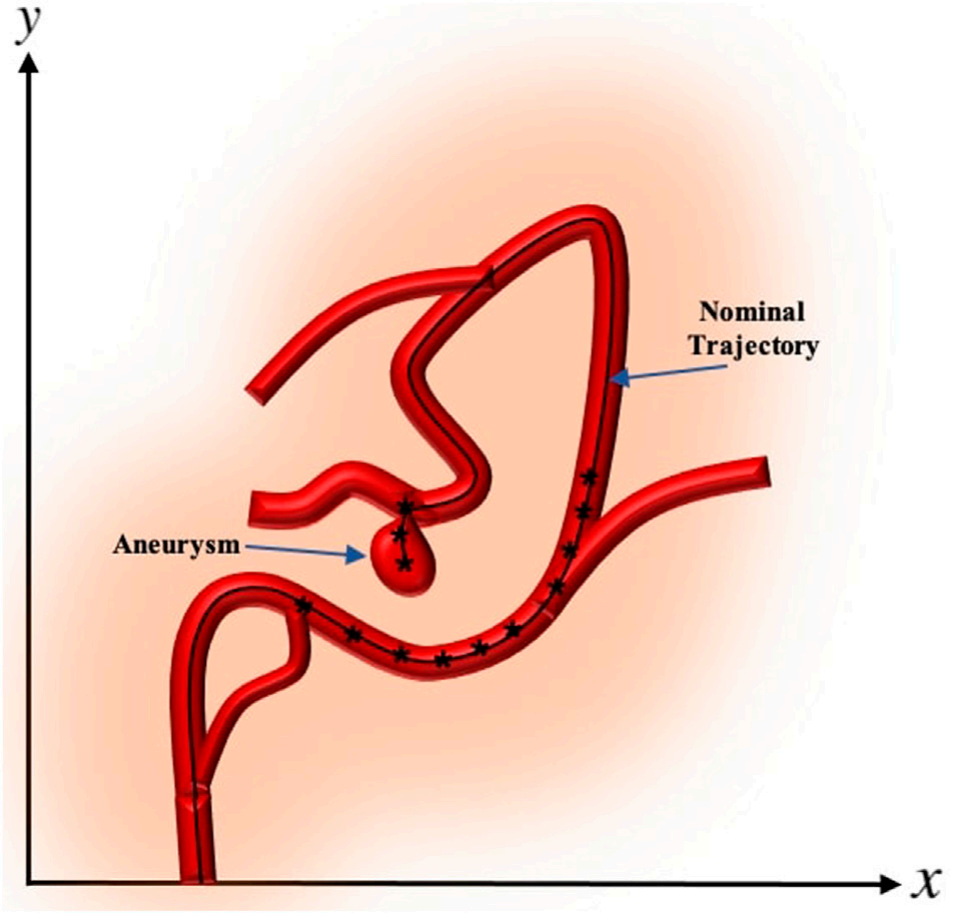
The nominal trajectory and the nominal points generated along a vessel with aneurysm.

The desired trajectory, also called the nominal trajectory, is the path that the catheter needs to pass in order to enter the branches which lead to the desired target location where aneurysm is located. Points need to be localized on the generated desired trajectory, which allow the catheter tip to reach the target location while avoiding unwanted collisions to the vessel walls. We consider each point along the nominal trajectory as the step-wise desired position of the tip of catheter and refer to these points as nominal points. The proximity, density, and location of the nominal points are adjusted according to the vessels’ anatomy and practical considerations O’Flynn et al. (2007). These considerations include the arterial bifurcations, sharpness of the change of angles along the trajectory, length of vascular branches, delicacy of veins, and many other factors that can be based on the expert’s knowledge. In some cases, the nominal trajectory is generated only near the bifurcations. Once the catheter is deflected and mechanically guided into the appropriate vessel branch, the moment can be removed and there will be no additional moment required to maintain the catheter position. This is analogous to existing catheter designs where the natural elasticity of the catheter tip, which would tend to restore native catheter tip geometry, will be offset by mechanical resistance from the vessel wall Roberts et al. (2002). An example of the nominal trajectory along a vessel with aneurysm and the nominal points generated on the trajectory are demonstrated in Figure 4. In the numerical experiments of this paper, without loss of generality, we consider the centerline of the vasculature leading to the aneurysm as the desired trajectory and generate points uniformly along the desired trajectory.

According to the generated nominal trajectory and the nominal points along the trajectory, the goal is to obtain the set of actions that a clinician can take to guide the catheter through the vasculature and reach the target location. By defining *t* as the current step where the catheter is located and *n* as the number of actuators along the catheter, we consider the additional moments applied by the actuators (Δ**M**) and the insertion depth (axial motion) of the catheter (Δ*d*) as the set of actions that can be taken at each step. Therefore, the set of actions at step *t* is represented in the action vector **u**_*t*_ as:

$$u_{t}=\left[\Delta M_{t}\;\;\;\;\Delta d_{t}\right],$$ (6)

where Δ**M**_*t*_ = [Δ*M*_1,*t*_, *…* , Δ*M*_*n*,*t*_] with Δ*M*_*i*,*t*_ being the additional moment applied by actuator *i* at step *t*, for *i* = 1, *…* , *n*.

Assuming that at step *t*, the catheter with initial coordinates $\left[x_{t}^{init},\;\;y_{t}^{init}\right]$ is under the moments **M**_*t*_ = [*M*_1,*t*_, *…* , *M*_*n*,*t*_], we need to find the position of any point on the catheter at step *t* + 1 by applying the actions in the action vector **u**_*t*_ defined in Eq. 6. Having the action moments Δ**M**_*t*_ exerted by the actuators, the total moments applied to the catheter at step *t* + 1 can be obtained as:

$$M_{t+1}=M_{t}+\Delta M_{t}.$$ (7)

By axial movement of the catheter along the trajectory with the insertion depth of Δ*d*_*t*_, and assuming that the angle of the initial point on the catheter with respect to the horizontal line at step *t*, i.e. $\theta_{t}^{init}$ , is known based on the preoperative scans, the coordinates of the catheter’s initial point at step *t* + 1 are obtained as:

$$\begin{array}{cc} & x_{t+1}^{init}=x_{t}^{init}+\Delta d_{t}\;\;\cos\left(\theta_{t}^{init}\right),\\ & y_{t+1}^{init}=y_{t}^{init}+\Delta d_{t}\;\;\sin\left(\theta_{t}^{init}\right). \end{array}$$ (8)

**FIGURE 5:**
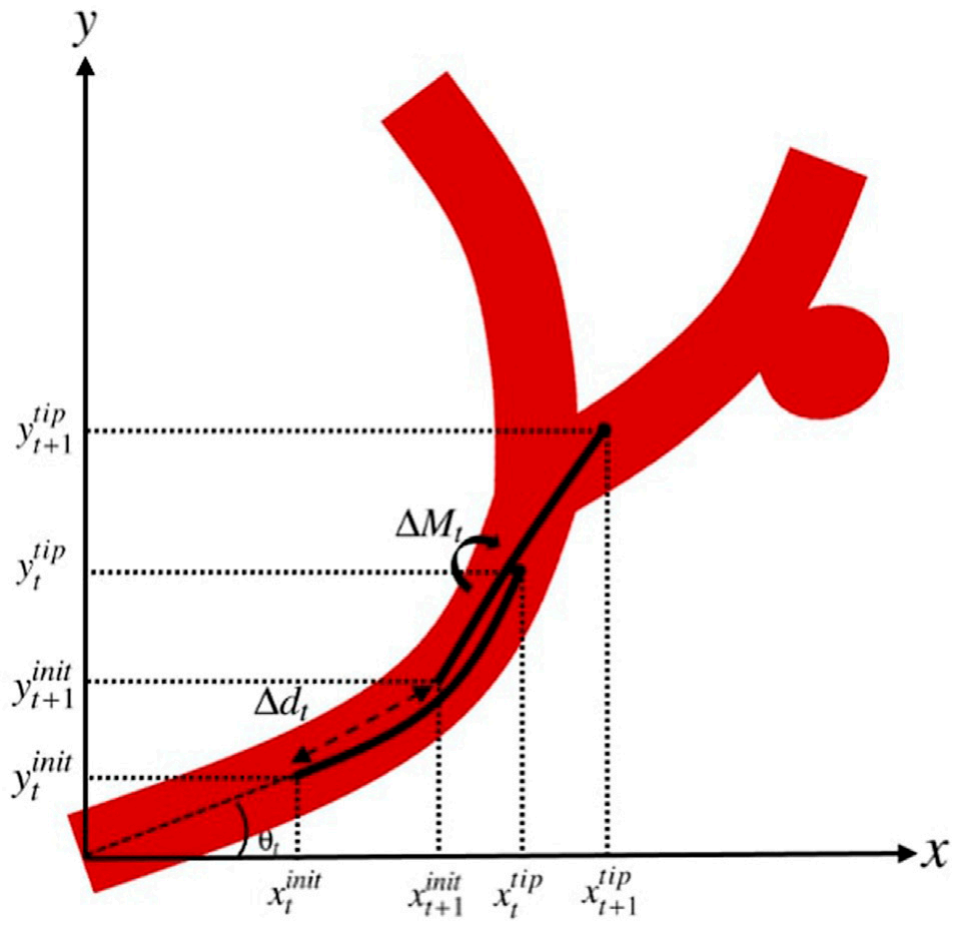
Actions applied to the catheter to guide it from the state at step *t* to *t* +1.

A demonstration of the actions applied to the catheter to guide it from the state at step *t* to *t* + 1 is represented in Figure 5.

We define the state vector $s_{t}=\left[M_{t}\;\;\;\;x_{t}^{init},\;\;y_{t}^{init},\;\;\theta_{t}^{init}\right]$ as the sufficient information to obtain the coordinates of any point on the catheter. Therefore, by having the knowledge about the state of catheter at step *t* and using Eqs 4–8, the coordinates of any point with distance *l* ^*s*^ from the initial point of the catheter at step *t* + 1 after applying **u**_*t*_ = [Δ**M**_*t*_, Δ*d*_*t*_] can be obtained as:

$$\begin{array}{cc} & x_{t+1}\left(u_{t},l^{s}\right)=f\left(s_{t},u_{t},l^{s}\right),\\ & y_{t+1}\left(u_{t},l^{s}\right)=g\left(s_{t},u_{t},l^{s}\right), \end{array}$$ (9)

where

$$\begin{array}{cc} f\left(s_{t},u_{t},l^{s}\right)= & x_{t}^{init}+\Delta d_{t}\;\;\cos\left(\theta_{t}^{init}\right)+\left(\overset{i-1}{\underset{j=1}{\sum }}\left[\left(\frac{EI}{M_{j,t}+\Delta M_{j,t}}-\frac{EI}{M_{j+1,t}+\Delta M_{j+1,t}}\right)\;\;\sin\left(\overset{j}{\underset{c=1}{\sum }}\frac{\left(M_{c,t}+\Delta M_{c,t}\right)\;l_{c}}{EI}\right)\right]\right.\\ & \left.+\frac{EI}{M_{i,t}+\Delta M_{i,t}}\sin\left(\frac{\left(M_{i,t}+\Delta M_{i,t}\right)\;l_{i}^{s}}{EI}+\overset{i-1}{\underset{j=1}{\sum }}\frac{\left(M_{j,t}+\Delta M_{j,t}\right)\;l_{j}}{EI}\right)\right)\times \cos\left(\theta_{t}^{init}\right)\\ & -\left(\frac{EI}{M_{1,t}+\Delta M_{1,t}}+\overset{i-1}{\underset{j=1}{\sum }}\left[\left(\frac{EI}{M_{j+1,t}+\Delta M_{j+1,t}}-\frac{EI}{M_{j,t}+\Delta M_{j,t}}\right)\;\;\cos\left(\overset{j}{\underset{c=1}{\sum }}\frac{\left(M_{c,t}+\Delta M_{c,t}\right)\;l_{c}}{EI}\right)\right]\right.\\ & \left.-\frac{EI}{M_{i,t}+\Delta M_{i,t}}\times \cos\left(\frac{\left(M_{i,t}+\Delta M_{i,t}\right)\;l_{i}^{s}}{EI}+\overset{i-1}{\underset{j=1}{\sum }}\frac{\left(M_{j,t}+\Delta M_{j,t}\right)\;l_{j}}{EI}\right)\right)\sin\left(\theta_{t}^{init}\right), \end{array}$$ (10)

and

$$\begin{array}{cc} g\left(s_{t},u_{t},l^{s}\right)= & y_{t}^{init}+\Delta d_{t}\;\;\sin\left(\theta_{t}^{init}\right)+\left(\overset{i-1}{\underset{j=1}{\sum }}\left[\left(\frac{EI}{M_{j,t}+\Delta M_{j,t}}-\frac{EI}{M_{j+1,t}+\Delta M_{j+1,t}}\right)\;\;\sin\left(\overset{j}{\underset{c=1}{\sum }}\frac{\left(M_{c,t}+\Delta M_{c,t}\right)\;l_{c}}{EI}\right)\right]\right.\\ & \left.+\frac{EI}{M_{i,t}+\Delta M_{i,t}}\sin\left(\frac{\left(M_{i,t}+\Delta M_{i,t}\right)\;l_{i}^{s}}{EI}+\overset{i-1}{\underset{j=1}{\sum }}\frac{\left(M_{j,t}+\Delta M_{j,t}\right)\;l_{j}}{EI}\right)\right)\times \sin\left(\theta_{t}^{init}\right)\\ & +\left(\frac{EI}{M_{1,t}+\Delta M_{1,t}}+\overset{i-1}{\underset{j=1}{\sum }}\left[\left(\frac{EI}{M_{j+1,t}+\Delta M_{j+1,t}}-\frac{EI}{M_{j,t}+\Delta M_{j,t}}\right)\;\;\cos\left(\overset{j}{\underset{c=1}{\sum }}\frac{\left(M_{c,t}+\Delta M_{c,t}\right)\;l_{c}}{EI}\right)\right]\right.\\ & \left.-\frac{EI}{M_{i,t}+\Delta M_{i,t}}\times \cos\left(\frac{\left(M_{i,t}+\Delta M_{i,t}\right)\;l_{i}^{s}}{EI}+\overset{i-1}{\underset{j=1}{\sum }}\frac{\left(M_{j,t}+\Delta M_{j,t}\right)\;l_{j}}{EI}\right)\right)\cos\left(\theta_{t}^{init}\right). \end{array}$$ (11)

The next section discusses trajectory tracking and movement of the multi-actuator soft catheter along a desired trajectory.

### 3.3 Trajectory Tracking of Multi-Actuator Soft Catheter

Here, we focus on the primary goal of catheterization which is obtaining the motion trajectories by the set of actions that a clinician can take to guide the catheter through the vasculature and reach the target location. We define $\left[x_{t+1}^{NP}\;\;\;\;y_{t+1}^{NP}\right]$ as the coordinates of the nominal point along the desired trajectory at step *t* + 1. Having a catheter with length *l* [*x*_*t*+1_ (**u**_*t*_, *l*), *y*_*t*+1_ (**u**_*t*_, *l*)] specifies the coordinates of the tip of catheter. We aim to find the optimal set of actions at each step that move the tip of catheter to the next nominal point designated on the trajectory while keeping the body of catheter closest to the nominal trajectory for avoiding unwanted collisions to the vessel walls. Therefore, the problem is formulated as:

$$\begin{array}{cc} u_{t}^{*} & =\underset{u\in U}{\mathrm{argmin}}\left[\underset{d_{t+1}^{tip}}{\underset{︸}{\sqrt{{\left(x_{t+1}^{NP}-x_{t+1}\left(u_{t},l\right)\right)}^{2}+{\left(y_{t+1}^{NP}-y_{t+1}\left(u_{t},l\right)\right)}^{2}}}}\right.\\ & \left.\;\;\;\;\;\;+\gamma \;\;\underset{d_{t+1}^{body}}{\underset{︸}{\int_{0}^{l}\sqrt{{\left(x_{t+1}^{NT}-x_{t+1}\left(u_{t},l^{s}\right)\right)}^{2}+{\left(y_{t+1}^{NT}-y_{t+1}\left(u_{t},l^{s}\right)\right)}^{2}}dl^{s}}}\right]\\ & =\underset{u\in U}{\mathrm{argmin}}\left[\sqrt{{\left(x_{t+1}^{NP}-f\left(s_{t},u_{t},l\right)\right)}^{2}+{\left(y_{t+1}^{NP}-g\left(s_{t},u_{t},l\right)\right)}^{2}}\right.\\ & \left.\;\;\;\;\;\;+\gamma \;\;\int_{0}^{l}\sqrt{{\left(x_{t+1}^{NT}-f\left(s_{t},u_{t},l^{s}\right)\right)}^{2}+{\left(y_{t+1}^{NT}-g\left(s_{t},u_{t},l^{s}\right)\right)}^{2}}dl^{s}\right]\\ & \;s.t.\;d_{t+1}^{tip}\;<\;d_{thld}^{tip}\\ & \;\;d_{t+1}^{body}\;<\;d_{thld}^{body} \end{array}$$ (12)

where U is the space of actions, *γ* is a weight coefficient, and $\left[x_{t+1}^{NT}\;\;\;\;y_{t+1}^{NT}\right]$ are the coordinates of the point along the nominal trajectory closest to the point with distance *l* ^*s*^ from the catheter’s initial point. The weight coefficient *γ* characterizes the relative importance of closeness of catheter body and catheter tip to the centerline, and it is set according to the sensitivity of the vein to the catheter tip and catheter body. The first term in Eq. 12, i.e., $d_{t+1}^{tip}$ , is the distance of the tip of catheter from the point corresponding to step *t* + 1 on the nominal trajectory, and the second term, i.e. $d_{t+1}^{body}$ , is the distance of the catheter body from the nominal trajectory. The actions obtained in this optimization problem are constrained to maintaining the maximum threshold distance $d_{thld}^{tip}$ of the tip of catheter from the nominal point, and the maximum threshold distance $d_{thld}^{body}$ of the catheter body from the nominal trajectory. The integral for computing $d_{t+1}^{body}$ is calculated by Monte Carlo (MC) approximation by generating *S* samples along the part of nominal trajectory that the catheter body is located. It should be noted that this integral can be computed using a grid. Thus, the problem in Eq. 12 is restated as:

$$\begin{array}{cc} u_{t}^{*} & \approx \underset{u\in U}{\mathrm{argmin}}\left[\underset{d_{t+1}^{tip}}{\underset{︸}{\sqrt{{\left(x_{t+1}^{NP}-f\left(s_{t},u_{t},l\right)\right)}^{2}+{\left(y_{t+1}^{NP}-g\left(s_{t},u_{t},l\right)\right)}^{2}}}}\right.\\ & \left.\;\;\;\;\;\;+\underset{d_{t+1}^{body}}{\underset{︸}{\frac{\gamma }{S}\overset{S}{\underset{j=1}{\sum }}\sqrt{{\left(x_{t+1}^{NT}-f\left(s_{t},u_{t},l_{j}^{s}\right)\right)}^{2}+{\left(y_{t+1}^{NT}-g\left(s_{t},u_{t},l_{j}^{s}\right)\right)}^{2}}}}\right]\\ & s.t.\;d_{t+1}^{tip}\;<\;d_{thld}^{tip}\\ & \;d_{t+1}^{body}\;<\;d_{thld}^{body} \end{array}$$ (13)

where $l_{j}^{s}$ is the distance of *j*th MC sample from the initial point of the catheter. This non-linear constrained optimization problem can be solved using any non-linear optimizers, including population-based evolutionary optimization techniques such as genetic algorithms used in this paper. For the next procedural phase, the optimized actions $u_{t}^{*}$ are executed by the interventionalist in order to reach the new catheter position. This optimization problem is solved for all points on the nominal trajectory until the trajectory is complete. The proposed framework can benefit the catheterization procedures by providing the interventionalists the capability to select the appropriate catheter prior to surgeries. By accounting for the manufacturability considerations and the complexities that arise by adding the number of actuators, the optimal number of actuators that allow the catheter to reach the aneurysm location while satisfying the constraints in Eq. 13 will be selected by the interventionalist for the catheterization procedure. This pre-operative catheter selection avoids multiple insertion attempts often made by the interventionalist at a single site which lead to a more invasive procedure and cause patient discomfort.

## 4 Numerical Experiments

In this section, computational experiments are conducted to demonstrate the catheter selection process according to the maneuverability that can be achieved through the multi-actuator soft catheter robots. In all the experiments, the moment of inertia of cross section around the natural axis, i.e., *I*, is fixed along the catheter, considering circular area with moment of inertia $I=\frac{\pi r^{4}}{4}$ . The optimization problems are carried out using genetic algorithm implemented in MATLAB and the weight coefficient *γ* is set to 1 in all the experiments, assuming that it is equally important to keep the body and tip of catheter far from the centerline. All experiments are performed on a personal computer with an Intel 8-Core Core i7 CPU (3.8 GHz) and 16-GB memory. Throughout the numerical experiments, the unit of all the parameters associated with length is in *cm* and the other units are in SI metric system.

### 4.1 Single-Trajectory Scenarios

**FIGURE 6:**
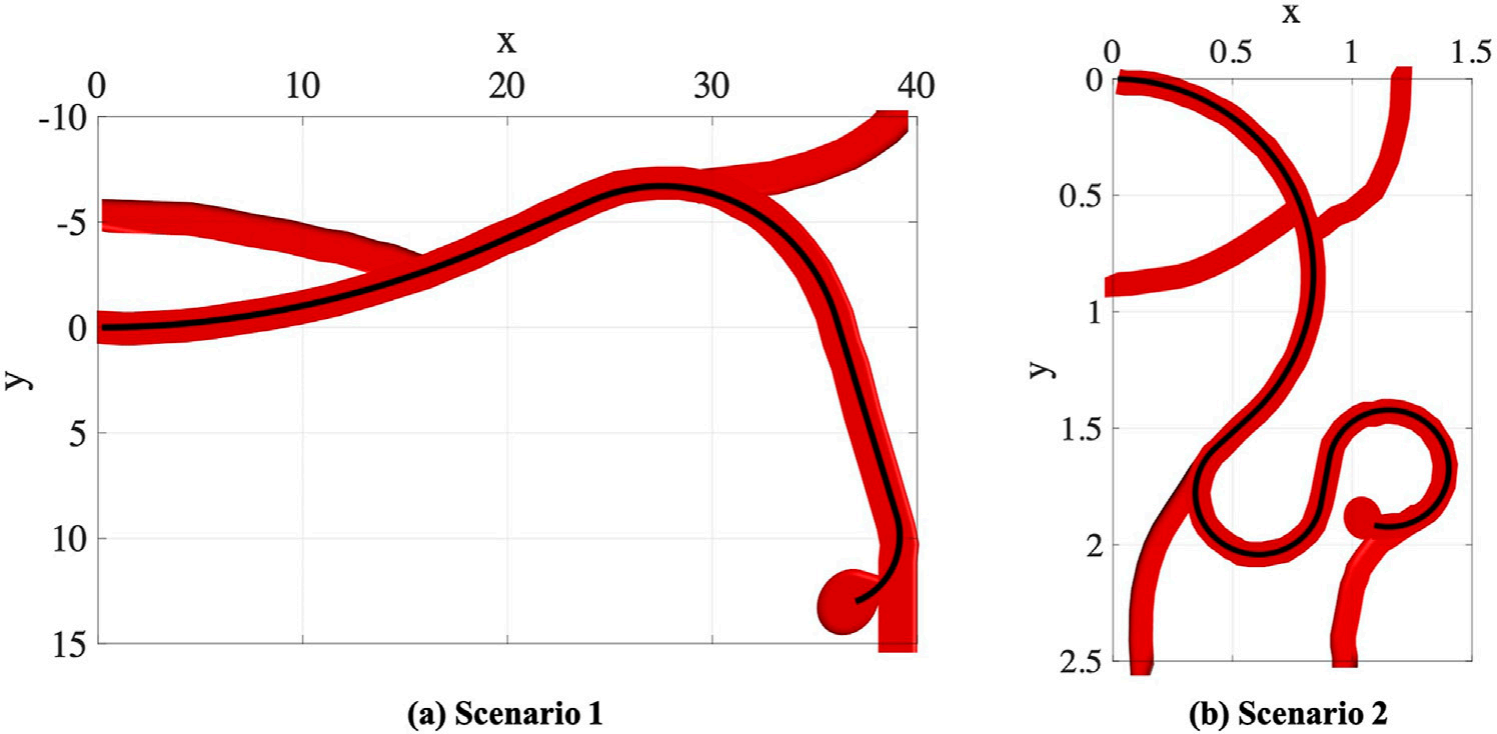
Simulated Single-Trajectory Scenarios (all dimensions are in *cm*).

In the first part of experiments, we consider two trajectory scenarios Ghoreishi et al. (2021) along the vessels with aneurysms as shown in Figure 6. These images demonstrate potential scenarios with increasing complexity and tortuosity. Scenario 1 demonstrates a small sidewall aneurysm arising from third order branch. Scenario 2 demonstrates added challenges due to increasing tortuosity and more distal location from fourth order branch vessel. However, the aneurysm axis is somewhat more in line with the vessel orientation.

#### 4.1.1 Scenario 1

**FIGURE 7:**
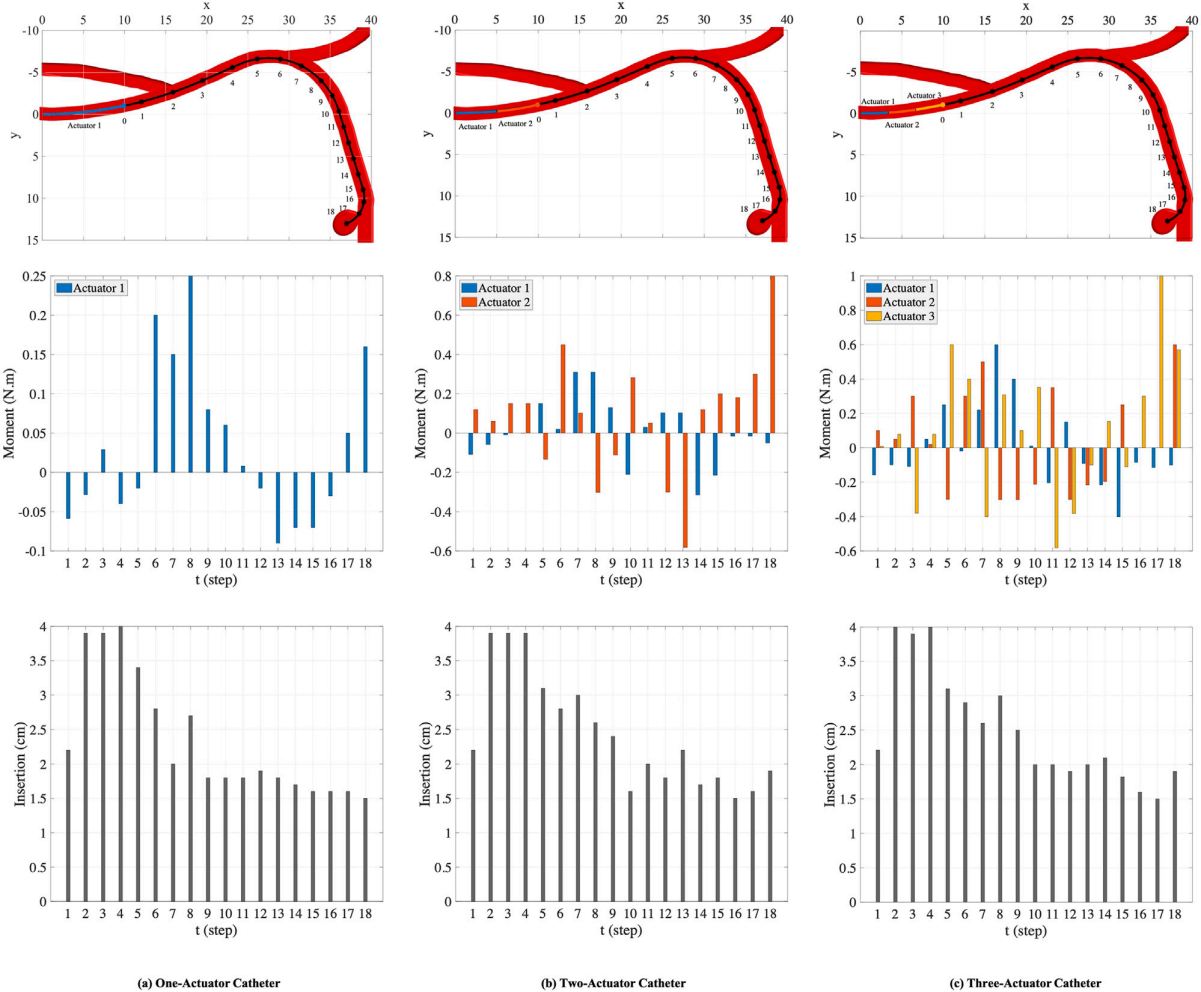
The nominal trajectory and the points generated on the trajectory **(top row)**. The moments **(middle row)** and the insertion depths **(bottom row)** applied by one-actuator, two-actuator, and three-actuator catheters in 18 steps.

In this scenario, we consider a circular catheter with the length of *l* = 10 *cm*, radius of *r* = 0.5 *cm*, and modulus of elasticity of *E* = 1 × 10^8^ *Pa*. The lengths of actuators along the catheter are assumed to be equal, i.e., the length of each actuator in an *n*-actuated catheter is $l_{i}=\frac{l}{n}$ , for *i* = 1, *…* , *n*. As shown in the top plots in Figure 7, catheters with one, two, and three actuators are considered for this scenario and 18 points are generated along the nominal trajectory. The number of MC samples for integral computation is set to *S* = 100. The space of actions that can be taken is set adaptively at each step. The range of moments that can be exerted by the actuators at step *t*, i.e., Δ**M**_*t*_, is set to $\left[\min\left\{-M_{\max},-|M_{i,t}|\right\}\;\;\;\;\;\max\left\{M_{\max},|M_{i,t}|\right\}\right]$ , where *M*_max_ is considered to be 1 *N*.*m* in this scenario, and the space of insertion depth of the catheter along the vessel is set to $\left[0.5\;d_{t}^{TN}\;\;\;\;\;2\;d_{t}^{TN}\right]$ , where $d_{t}^{TN}$ is the distance between the tip of catheter at current step and the next nominal point, i.e., $\left[x_{t}^{tip}\;\;\;\;y_{t}^{tip}\right]$ and $\left[x_{t+1}^{NP}\;\;\;\;y_{t+1}^{NP}\right]$ . The maximum threshold distances in the constraints are set to $d_{thld}^{tip}=0.5$ and $d_{thld}^{body}=2$ both in *cm*. The rationale behind setting $d_{thld}^{tip}$ smaller than $d_{thld}^{body}$ is the sharpness of the tip of catheter which can cause serious damage to the vessel walls if it gets very far from the nominal point at each step. However, more tolerance is considered for maximum distance of the catheter body from the nominal trajectory due to the natural resistance and elasticity of the vessel wall toward the blunt catheter body.

**FIGURE 8:**
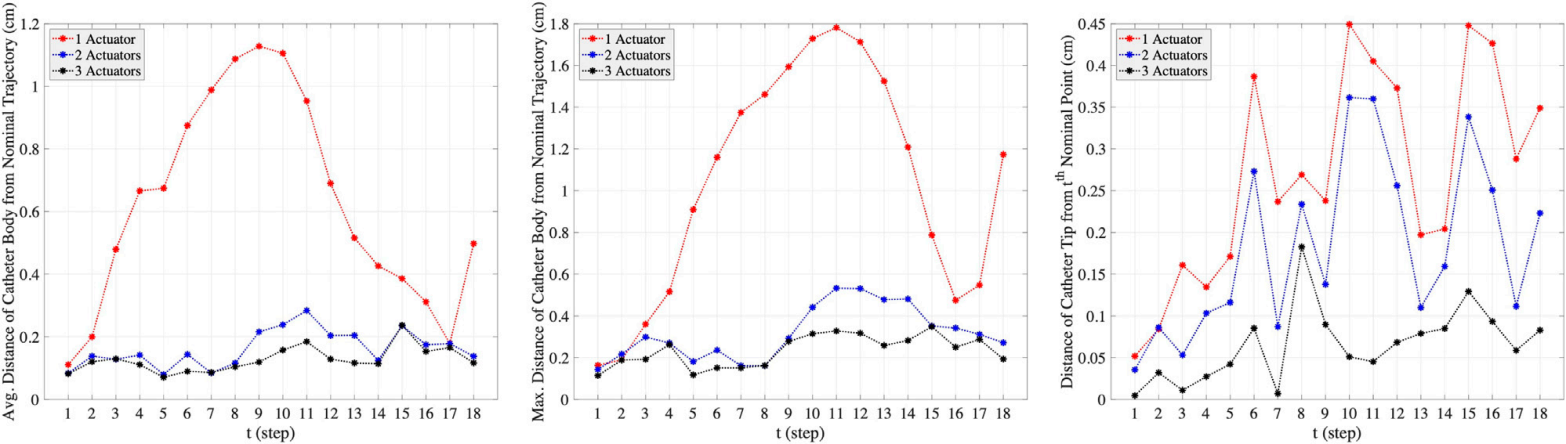
The average and maximum distance of catheter body from the nominal trajectory and the distance of catheter tip from the nominal points, achieved by one-actuator, two-actuator, and three-actuator catheters in tracking the trajectory scenario 1. The maximum threshold distances in the constraints are $d_{thld}^{body}=2\;cm$ and $d_{thld}^{tip}=0.5\;cm$ .

The plots in the columns (a), (b), and (c) in Figure 7 are associated with one-actuator, two-actuator, and three-actuator catheters respectively. The plots in the middle row represent the moments applied by each actuator and the plots in the bottom row show the insertion depth of catheter along the vessel at each step. It can be seen that the insertion depths in all three catheters are almost in the same range. However, the moment exerted by each actuator is different in catheters with one, two, and three actuators. The number of actuators and the moments applied by each actuator play the key role in performance of catheters in tracking the desired trajectory and reaching the target location where aneurysm is located. This is demonstrated in Figure 8, which represents the average and maximum distance of catheter body from the nominal trajectory and the distance of catheter tip from the nominal points, achieved by one-actuator, two-actuator, and three-actuator catheters at each step. The figure shows that the three-actuator catheter has the highest performance in following the desired trajectory as it maintains the smallest distance from the nominal trajectory and the nominal points compared to the catheters with one and two actuators. The two-actuator catheter performs reasonably well in this scenario, however the only actuator in the one-actuator catheter provides very limited flexibility which results in large distances while acceptable according the considered thresholds in the constraints.

#### 4.1.2 Scenario 2

**FIGURE 9:**
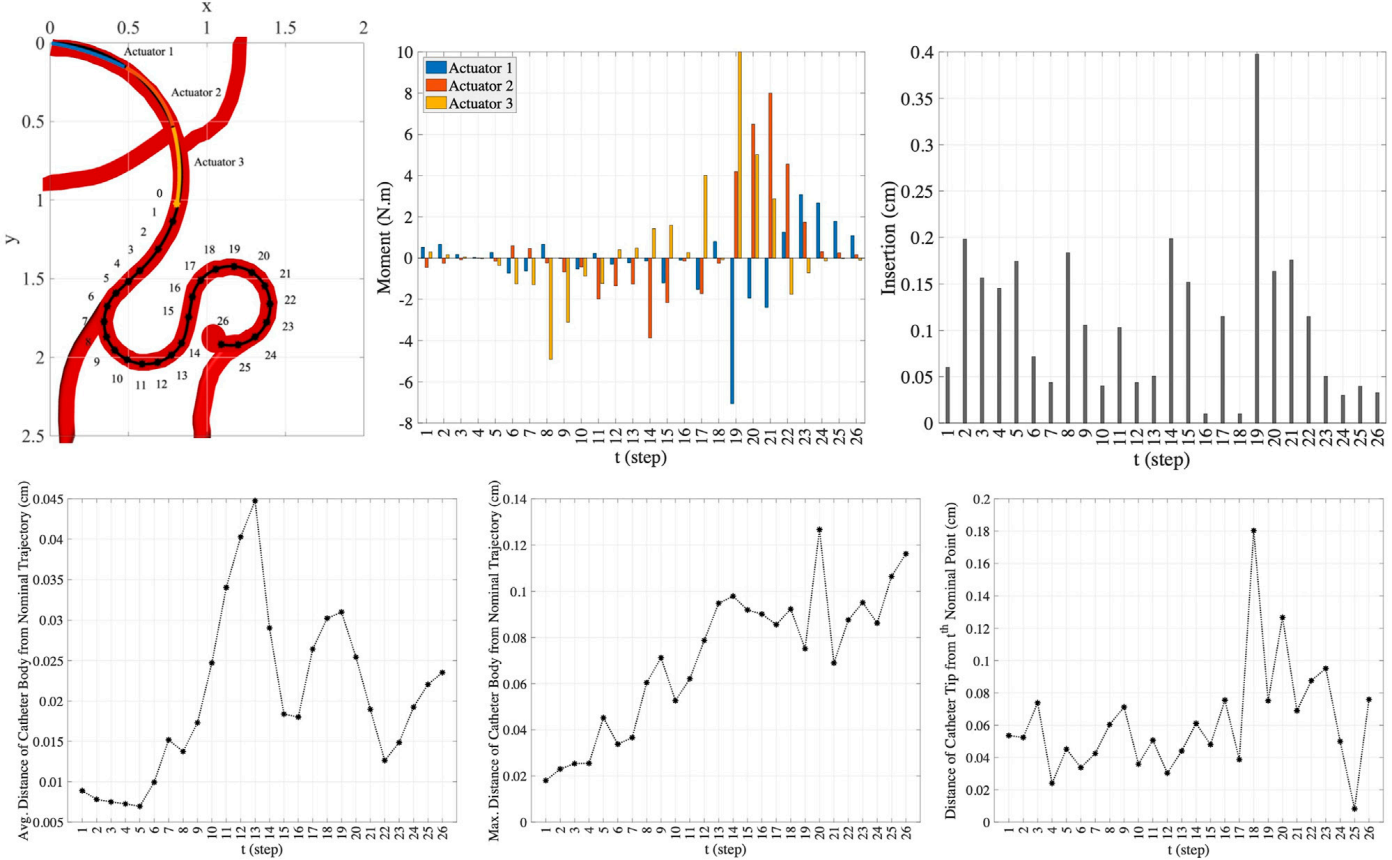
The nominal trajectory and the moments and the insertion depth applied by three-actuator catheter in 26 steps **(top row)** and the precision achieved in this trajectory tracking **(bottom row)**.

Here, we consider a more complex scenario in smaller scale in comparison to the trajectory scenario in the previous experiment. In this scenario, the geometric and material properties of the circular catheter are set to be *l* = 1.5 *cm*, *r* = 0.4 *cm*, and *E* = 1 × 10^8^ *Pa*. Like the previous experiment, the lengths of actuators along the catheter are assumed to be equal. As shown in the top left plot in Figure 9, we consider 26 points along the nominal trajectory. The number of MC samples for integral computation is set to *S* = 100. The space of actions that can be taken in order to guide the catheter from the current step to the next step is set adaptively at each step. The range of moments that can be exerted by the actuators at step *t*, i.e., Δ**M**_*t*_, is set to $\left[\min\left\{-M_{\max},-|M_{i,t}|\right\}\;\;\;\;\;\max\left\{M_{\max},|M_{i,t}|\right\}\right]$ , where *M*_max_ is considered to be 10 *N*.*m* in this scenario, and the space of insertion depth of the catheter along the vessel is set to $\left[0.5\;d_{t}^{TN}\;\;\;\;\;2\;d_{t}^{TN}\right]$ , where $d_{t}^{TN}$ is the distance between the tip of catheter at current step and the next nominal point, i.e., $\left[x_{t}^{tip}\;\;\;\;y_{t}^{tip}\right]$ and $\left[x_{t+1}^{NP}\;\;\;\;y_{t+1}^{NP}\right]$ . The maximum threshold distance of the catheter tip from the nominal point and the threshold distance of the catheter body from the nominal trajectory are respectively set to $d_{thld}^{tip}=0.2$ and $d_{thld}^{body}=0.5$ both in *cm*. These threshold distances are set smaller than those in the previous experiment due to the delicacy of the veins in this scenario compared to scenario 1.

In this scenario, the one-actuator and two-actuator catheters are unable to provide the adequate maneuverability to follow the desired trajectory as the distances from the target points and the nominal trajectory exceed the maximum threshold distances. However, the three-actuator catheter is capable of tracking this trajectory scenario by maintaining the constraints. The moments exerted by each of the three actuators and the insertion depth of the catheter at each step to move the catheter to the next step till reaching the target aneurysm location are demonstrated in the two top right plots in Figure 9. The plots in the bottom row in this figure show the average and maximum distance of the catheter body from the nominal trajectory as well as the distance of the catheter tip from the nominal point. It can be seen that the maximum threshold distances are satisfied at each step. This is achieved through the use of three actuators along the catheter, increasing the flexibility and maneuverability of the catheter. This indicates the benefit of selection of the number of actuators prior to endovascular catheterization procedures, which results in preventing the difficulties that can arise due to inappropriate selection of catheters with limited maneuverability.

### 4.2 Multi-Trajectory/Fractal Tree Scenarios

**FIGURE 10:**
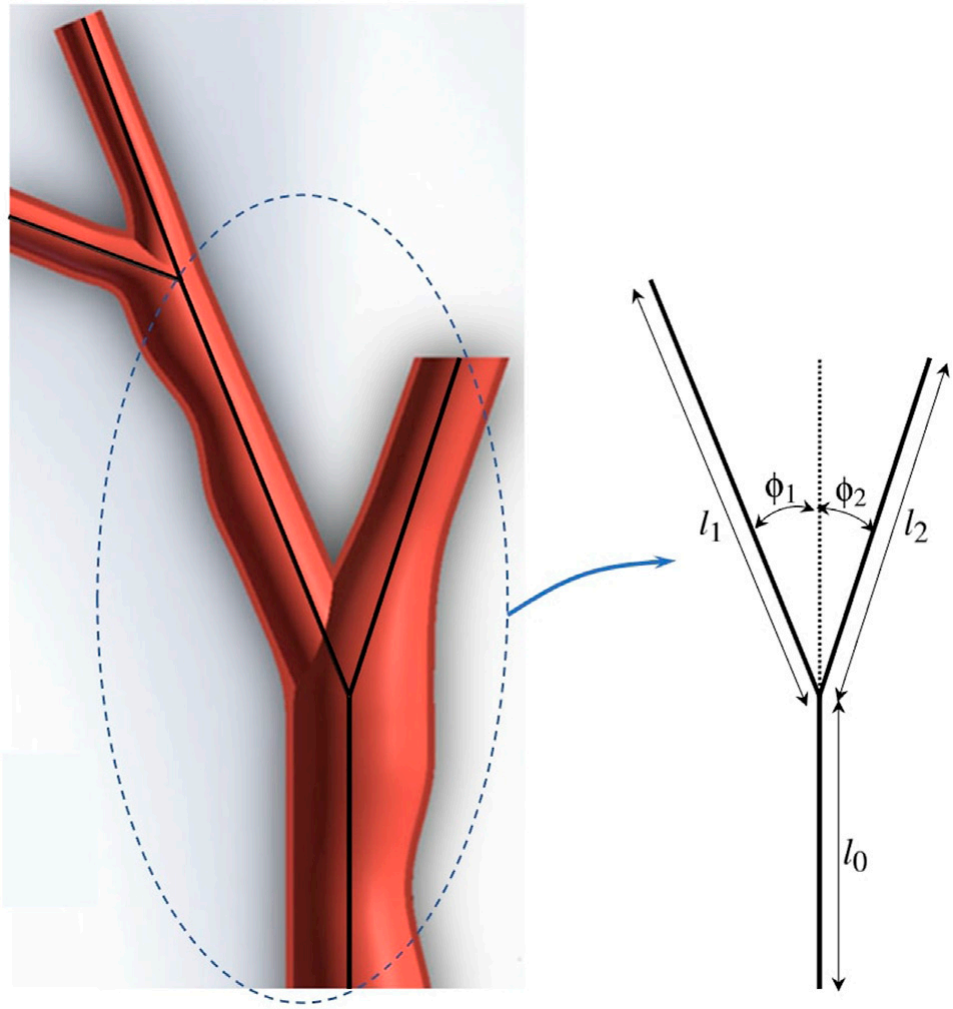
Fractal tree modeling of the vascular system.

**FIGURE 11:**
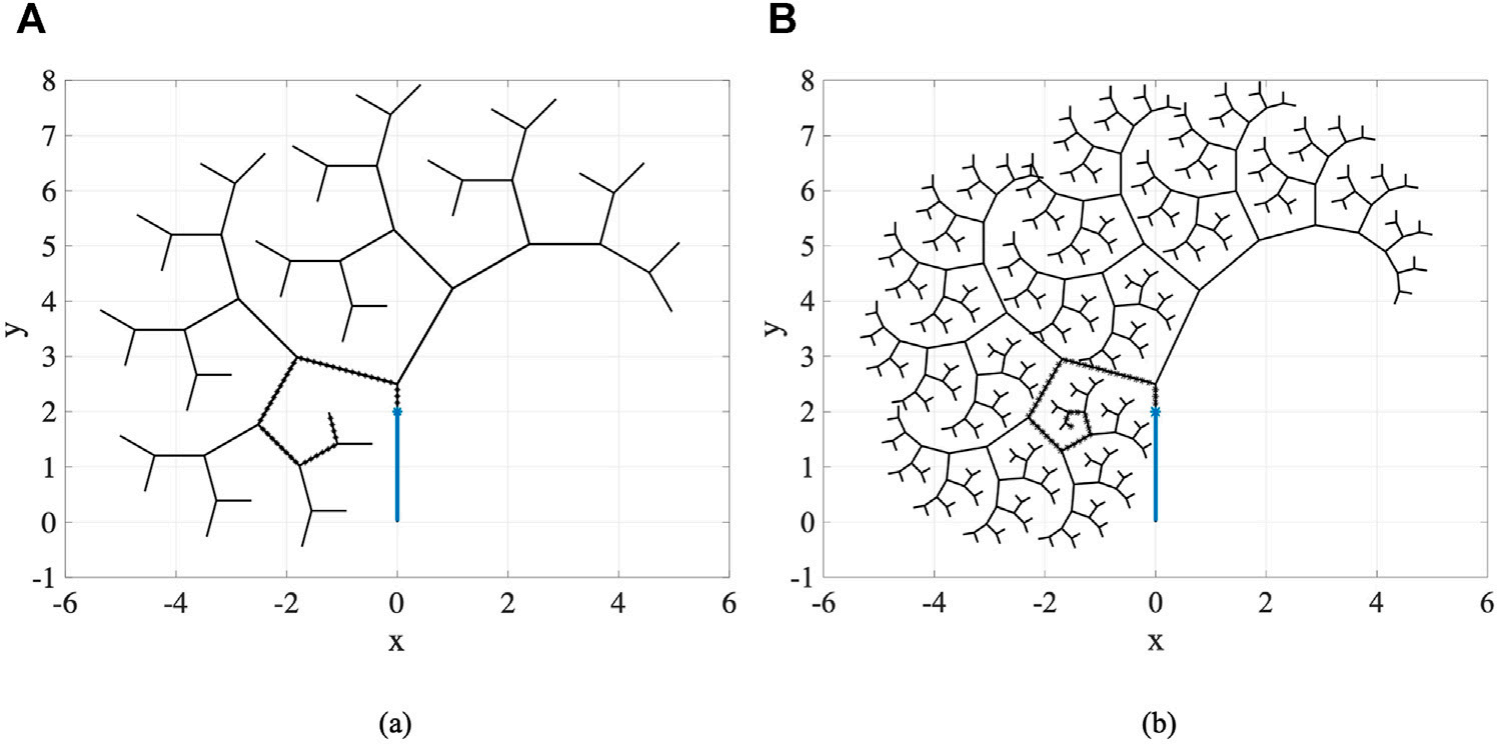
Fractal trees with parameters (a) $n_{br}=6,\;\;\;\lambda_{1}=0.75,\;\;\;\lambda_{2}=0.8,\;\;\;\phi_{1}=\frac{5\pi }{12},\;\;\;\phi_{2}=-\frac{\pi }{6}\;\;$ and (b) $n_{br}=9,\;\;\;\lambda_{1}=0.7,\;\;\;\lambda_{2}=0.75,\;\;\;\phi_{1}=\frac{5\pi }{12},\;\;\;\phi_{2}=-\frac{5\pi }{36}$ .

In this part of experiments, we consider fractal trees due to their similarity with branching structure patterns of arteries in vascular system Perdikaris et al. (2015). These fractal trees are assumed to be the vessel centerlines. To generate the fractal tree structures, we consider two branches stemming from each parent branch. We specify the number of branches *n*_*br*_ in a chain, the branching angles *ϕ*_1_ and *ϕ*_2_, and the length ratios $\lambda_{1}=\frac{l_{1}}{l_{0}}$ and $\lambda_{2}=\frac{l_{2}}{l_{0}}$ as the ratio of the length of branches to the length of the parent vessel at the bifurcation, as shown in Figure 10 for *n*_*br*_ = 2. In our computational analysis, we consider two fractal trees with parameters $n_{br}=6,\;\;\lambda_{1}=0.75,\;\;\lambda_{2}=0.8,\;\;\phi_{1}=\frac{5\pi }{12},\;\;\phi_{2}=-\frac{\pi }{6}$ and $n_{br}=9,\;\;\lambda_{1}=0.7,\;\;\lambda_{2}=0.75,\;\;\phi_{1}=\frac{5\pi }{12},\;\;\phi_{2}=-\frac{5\pi }{36}$ as in Ajam et al. (2017), which we show in Figure 11.

For this set of experiments, the geometric and material properties of the circular catheter are considered to be *l* = 2 *cm*, *r* = 0.4 *cm*, and *E* = 1 × 10^8^ *Pa*, indicating the length, radius, and modulus of elasticity of the catheter respectively. For all the generated branches and considering each sequence of branches one at a time as the desired trajectory, we perform our proposed framework for different number of actuators with equal lengths of $l_{i}=\frac{l}{n}$ for *i* = 1, *…* , *n*, where *n* is the number of actuators. We consider uniform points along the nominal trajectories to follow by the catheter, as shown for one desired trajectory of the fractal trees in Figure 11. Similar to the previous experiments, the space of actions to guide the catheter from the current step to the next step is set adaptively at each step. The range of moments that can be exerted by the actuators at step *t*, i.e., Δ**M**_*t*_, is set to $\left[\min\left\{-M_{\max},-|M_{i,t}|\right\}\;\;\;\;\;\max\left\{M_{\max},|M_{i,t}|\right\}\right]$ , where *M*_max_ is considered to be 5 *N*.*m* in these scenarios, and the space of insertion depth of the catheter along the vessel is set to $\left[0.5\;d_{t}^{TN}\;\;\;\;\;2\;d_{t}^{TN}\right]$ , where $d_{t}^{TN}$ is the distance between the tip of catheter at current step and the next point along the nominal trajectory. The maximum threshold distance of the catheter tip from the nominal point and the maximum threshold distance of the catheter body from the nominal trajectory are set to $d_{thld}^{tip}=0.1\;cm$ and $d_{thld}^{body}=0.2\;cm$ . The number of MC samples for integral computation is set to *S* = 100.

Table 1 represents the results averaged over all trajectories in the fractal trees shown in Figure 11. In this table, the success rate, average computation time, and average discrepancy from the centerline are presented for different number of actuators, where success rate is the percentage of the branches in a fractal tree that catheter could follow completely. According to the percentages of success rate, it can be seen that the three- and four-actuator catheters are able to successfully follow all the trajectories in fractal tree (a). However, considering the control and design complexities that arise by increasing the number of actuators, it is desired to select the three-actuator catheter for this fractal tree. In fractal tree (b), only the four-actuator catheter is capable of tracking all the trajectories while satisfying the threshold distance constraints. It can be seen that as the number of actuators increases, the average discrepancy of the catheter body from the centerlines decreases in both fractal trees; although the average discrepancies are higher in fractal tree (b) due to its complexity. The small average computation time of the proposed framework for trajectory tracking in these fractal trees emphasizes the benefits that can be achieved by pre-operative selection of proper catheters, avoiding the significant time, cost, and risk of catheterization procedures if inappropriate catheters are selected.

**TABLE 1:** Results averaged over branches of fractal trees in Figure 11 obtained for catheters with different number of actuators.

Fractal tree parameters	Number of actuators	Success rate (%)	Average computation time (s)	Average discrepancy from centerline (cm)
*n*_*br*_ = 6	1	25.0917	567.6153	0.0738
*λ*_1_ = 0.75 *λ*_2_ = 0.8	2	68.7500	572.3809	0.0564
$\phi_{1}=\frac{5\pi }{12}\;\phi_{2}=-\frac{\pi }{6}$	3	100	602.3808	0.0275
	4	100	774.7404	0.0247
*n*_*br*_ = 9	1	21.8750	710.3384	0.1288
*λ*_1_ = 0.7 *λ*_2_ = 0.75	2	62.5000	844.6492	0.0678
$\phi_{1}=\frac{5\pi }{12}\;\phi_{2}=-\frac{5\pi }{36}$	3	99.2188	1,078.6013	0.0389
	4	100	1,308.1174	0.0261

## 5 Conclusion

Interventional medicine is seeing a growing trend toward minimally invasive and catheter-based therapy, including in cerebrovascular procedures for treatment of cerebral aneurysms. Catheter-based surgeries can decrease hospitalization time and greatly lower patient morbidity compared to traditional open methods. However, catheter-based surgeries are often hindered by the lack of maneuverability of conventional catheters. Maneuverability of a catheter for intravascular navigation is a key to reaching the target area and it affects to a great extent the length and success of the procedure. This paper provided a simulation-based framework for pre-operative selection of catheters with desired level of maneuverability for treatment of cerebral aneurysm. The desired maneuverability is achieved by considering the appropriate number of pneumatic actuators along the catheter. The formulations for static deflection and dynamic modeling of multi-actuator soft catheter for trajectory tracking in two dimensions are provided.

Future work includes the intertwined design and dynamic analysis of multi-actuator soft catheters in three-dimensional space. In this work, the shear forces between the catheter body and blood which are mainly important in dynamic analysis of catheters, are not considered. Further, the contact forces between the catheter and vessel walls are modeled by the tolerance of the vessel walls in keeping the catheter inside the vessels, accounted by the maximum threshold distance of the tip of catheter from the nominal point and the maximum threshold distance of the catheter body from the nominal trajectory. These contact forces play a critical and unavoidable role in catheterization procedures. Thus, the interactions of catheter with blood and vessel walls and the resultant contact forces need to be studied extensively in future research to model realistic catheterization scenarios.

## Data Availability

The original contributions presented in the study are included in the article/Supplementary Material, further inquiries can be directed to the corresponding author.
